# JC Polyomavirus T-antigen protein expression and the risk of colorectal cancer: Systematic review and meta-analysis of case-control studies

**DOI:** 10.1371/journal.pone.0283642

**Published:** 2023-03-31

**Authors:** Lenka J. Kimla, Taane G. Clark, Sri Banerjee, Susana Campino

**Affiliations:** 1 Faculty of Infectious and Tropical Diseases, London School of Hygiene and Tropical Medicine, London, United Kingdom; 2 Walden University, Minneapolis, Minnesota, United States of America; 3 Faculty of Epidemiology and Population Health, London School of Hygiene and Tropical Medicine, London, United Kingdom; 4 Ross University School of Medicine, Miramar, Florida, United States of America; CNR, ITALY

## Abstract

JC Polyomavirus (JCV) is a human polyomavirus encoding T-antigen protein, which is implicated in carcinogenesis. JCV is prevalent in the upper and lower gastrointestinal track. Several studies have reported JCV associations with the risk of developing colorectal cancer (CRC), however, these findings remain controversial. Since JCV DNA may be present in healthy tissues as well as transformed tissues, JCV T-antigen expression could be a more useful measure of JCV’s association with cancer development. The aim of this study is to conduct a meta-analysis of case-control studies to investigate if there is a significant association between JCV T-antigen protein expression and risk of CRC. A systematic review was performed to identify studies reporting JCV DNA prevalence in CRC and JCV T-antigen expression. The strength of the association was estimated by odds ratios (ORs). Five (of 66) studies satisfied analysis inclusion criteria, and spanned years 1999 to 2022. Random effects meta-analysis of CRC cases versus controls showed an 11-fold increased risk of CRC development in JCV DNA positive samples with JCV T-antigen expression versus normal tissues (OR 10.95; 95% CI: 2.48–48.24; P = 0.0016). The results of this meta-analysis of JCV infection followed by JCV T-antigen protein expression for the risk of CRC support the argument that JCV infection significantly increases the risk of colorectal cancer in tissues where the JCV T-antigen protein is expressed. Further research with JCV T-antigen expression in relation to CRC development is needed.

## Introduction

Colorectal cancer (CRC) is the second leading cause of cancer death and the third most common cancer diagnosis worldwide [1, 2]. Various bacterial and viral agents have been implicated in the development of several cancers, including colorectal [3–5]. However, progress in establishing infectious origin of human cancers has been slow and controversial. Particularly well established is the causal association between human papillomavirus (HPV) and cervical cancer, Merkel cell virus causing 80% of skin cancer aka Merkel cell carcinoma, and the human T-cell lymphotropic virus type 1 (HTLV-1) with a rare form of leukemia [6–9]. The estimate of cancers being caused by an infectious agent has risen from 16 to 20% in the past 10 years, with marked increases related to the gastrointestinal tract [10, 11]. *Helicobacter pylori* and Epstein-Barr virus have been confirmed as causative pathogens in gastric cancer and, recently, *H*. *pylori* has been reported to increase the risk of CRC [3, 5, 12–14]. Establishing the potential role of other pathogens in cancer development is an active area of research. Organisms that have been implicated include bacteria such as; *Streptococcus bovis/gallolyticus*, *Escherichia coli*, *Bacteroides*, and viruses including; cytomegalovirus, human herpesviruses, human bocavirus, HPV, Inoue-Melnick virus, the polyoma viruses, simian virus 40 (SV40) and the JC Polyomavirus (JCV) [3–5]. Polyoma viruses, such as JCV, are remarkable for their ancient evolutionary origin, highly conserved genes, and encoding the large T (transformation)-antigen thought to be the most powerful of all transforming genes [15, 16]. The JCV T-antigen protein is capable of binding and inactivating the tumour suppressor proteins p53 and pRb, thus promoting carcinogenesis [16].

JCV was first discovered in 1971 in a patient with progressive multifocal leukoencephalopathy (PML) [17]. This polyomavirus is a small 5.13 kb closed, non-enveloped, circular, negatively supercoiled double stranded (ds) DNA virus with only six genes that form a mini chromosome with cellular histones, which they acquire only in infected cells [16, 18–21]. It exists as an episome, and at some point during infection, JCV DNA incorporates into human DNA [21–26]. Its oncogenic potential is thought to be due to the expression of the viral early T-antigen, which can affect the p53 tumour suppressor protein expression, cause chromosomal instability, and interaction with beta-catenin [20, 27, 28]. The interaction and nuclear localization of the T-antigen with both p53 and beta-catenin lead to oncogenesis, causing the uncontrolled proliferation of cells through a suppressed apoptosis and a cascade of upregulated gene expression [27].

JCV antibodies are present in up to 90% of the general adult population except for secluded populations in South America and Papua New Guinea [29–33]. JCV is usually acquired early in life, most probably via fecal-oral route, and causes a silent, lifelong infection, which is latent in the kidneys, central nervous system (CNS) and CD34+ lymphocytes [29–31, 34, 35]. While the majority of JCV infections are asymptomatic, the virus reactivates during an immunosuppressed state [9]. The virus is highly neurotropic and in JCV permissive cells, the oligodendrocytes, causes a lytic infection culminating in PML in immunosuppressed individuals [9, 30, 32]. JCV has been found, with some controversy, to be associated with glial-derived brain tumors [9, 22, 36, 37]. JCV is highly prevalent in the upper and lower gastrointestinal tract of immunocompetent individuals [34]. In JCV non-permissive cells (not permitting cell lysis), like the colon mucosa where JCV is found in high frequency, re-activated JCV may be associated with the development of adenomas and CRC [18, 38, 39]. Liver transplant recipients (LTR) undergoing an immunosuppressive treatment prior to their transplants have a higher rate of CRC development and a higher frequency of JCV DNA in CRC cases compared with controls [40]. A 2009 review of available data indicated that JCV fulfills almost all criteria necessary to establish a causal relationship between JCV and cancer, similar to HPV [9]. These criteria included detection of viral genome or gene products in cancer tissue, a molecular basis for virus-induced oncogenicity, and consistency of the association, and it has been highlighted that epidemiological studies linking JCV to human cancers were lacking [9, 41].

Meta-analyses have been conducted on named viruses and bacteria, demonstrating an association between infection and carcinogenesis [7–9, 42–46]. Recently published meta analyses on JCV infection and the risk of CRC showed that JCV increases the risk of CRC by 2-fold (P = 0.0008; compared to matched normal tissues) and 4.5-fold (P < 0.0001; compared to non-CRC healthy controls). Another systematic review demonstrated that the presence of JCV in colorectal tissues increased the risk of colorectal cancer by 4.70 times (OR Pooled = 4.70; 95% CI: 2.95–7.50) [47–49]. Since JCV DNA may be present in healthy tissues as well as transformed tissues, JCV T-antigen protein expression (used interchangeably with T-antigen expression) may be more important measure of JCV’s association with cancer development than the presence of T-antigen JCV DNA only [40]. However, no meta-analysis has been conducted on published case-control studies of JCV T-antigen expression and the risk of CRC in JCV DNA positive tissues. Here we aim to perform a meta-analysis to assess the link between JCV T-antigen expression and the risk of CRC using data from case-control studies using PCR for viral DNA detection carried out in CRC and normal tissues that also analyzed the expression of JCV T-antigen using immunohistochemistry (IHC) in JCV DNA positive tissues in cases and controls.

## Methods

### Literature search strategy

We conducted a systematic literature search, according to PRISMA (Preferred Reporting Items for Systematic Reviews and Meta-Analyses) guidelines, to identify publications that study the presence of JCV infection in the tissues of colorectal cancer patients and controls [50, 51]. We searched PubMed, Medline, CINAHL, ScienceDirect, Academic Search Complete, and Gale Academic OneFile Select for studies published between January 1, 1999 to March 27, 2022 with the following search terms: (((JC virus[Title/Abstract] OR JCV[Title/Abstract]) OR polyomavirus[Title/Abstract]) AND colorectal[All Fields]) AND ("neoplasms"[MeSH Terms] OR "neoplasms"[All Fields] OR "cancer"[All Fields]) AND ("1999/01/01"[PDAT]: "2022/03/27"[PDAT]). A supplemental bibliographic review of primary references was also conducted.

### Selection and inclusion criteria

Titles, abstracts, and methods sections of records were reviewed to identify case-control studies published in English between January 1, 1999 and March 27, 2022 that used PCR, with or without topoisomerase I treatment, to detect the presence of JCV T-antigen DNA and/or studies that conducted an IHC assay to detect the expression of JCV T-antigen protein in tissues of patients with colorectal cancer to include in the analysis.

Two types of case-control studies were eligible that conducted IHC tissues with confirmed JCV DNA in cases and controls (i) studies comparing CRC or adenoma cases with matched adjacent or distal healthy tissues from the same patients, and (ii) studies comparing CRC or adenoma cases with healthy controls that had normal colonoscopy or colonic biopsies (without cancer lesions). Studies were excluded if they were (1) expert reviews or systematic reviews, (2) case only studies, (3) studies without reporting on JCV DNA presence and/or IHC staining in cases and controls, (4) studies conducting JCV DNA analysis on tissues by techniques other than PCR, (5) seropositivity studies or studies conducted on samples other than tissue.

### Data extraction and quality assessment

Two independent reviewers confirmed the data accuracy and conflicts were resolved by consensus [50, 51]. Extracted data included first authors’ name, the year of publication, country of patient’s origin, type of neoplasm for cases, type of controls, patients mean age and range, molecular analyses and test methods, sample DNA integrity control, topoisomerase I treatment, JCV targeted DNA region for PCR, sample type (Paraffin embedded tissue (PET) or Fresh frozen tissue (FFT)), the number of JCV T-antigen DNA positive and negative individuals in cases and controls, the number of individuals with JCV T-antigen protein expression in their positive JCV T-antigen DNA samples, viral loads, and adjusted or crude OR with 95% CI. The quality of each study was evaluated by the Newcastle-Ottawa scale for case-control studies in which a study is rated by a ‘star system’ for a score of 1–10 on three broad perspectives [52].

### Statistical analysis

The strength of association between JCV infection with T-antigen expression and CRC was estimated by odds ratios (ORs) and their 95% confidence intervals (CI), calculated using the MedCalc software (v20.114) by adding 0.5 units to all 2*2 tables [53]. The significance of the (pooled) OR was calculated by the Z test, with p<0.05 considered to be statistically significant. Heterogeneity was estimated by the Cochrane-Q test and I-squared test statistics, with p>0.05 and I-squared > 25% indicating evidence of heterogeneity (I-squared >25% = low; >50% = medium and >75% = high heterogeneity) [54]. If heterogeneity was present random-effects model was used, if the effects lacked heterogeneity the fixed-effects model was also used according to the Mantel-Haenszel and DerSimonian-Laird methods [54–56]. Analysis was conducted by confirmed histologic type in cases (CRC and adenomas) and controls (matched adjacent normal). Meta-analyses were conducted using the R package metaphor [57]. Funnel plots were used to assess publication bias and the symmetry of the plot was assessed visually for asymmetry [57–59].

## Results

### Characteristics of studies included

**Fig 1:**
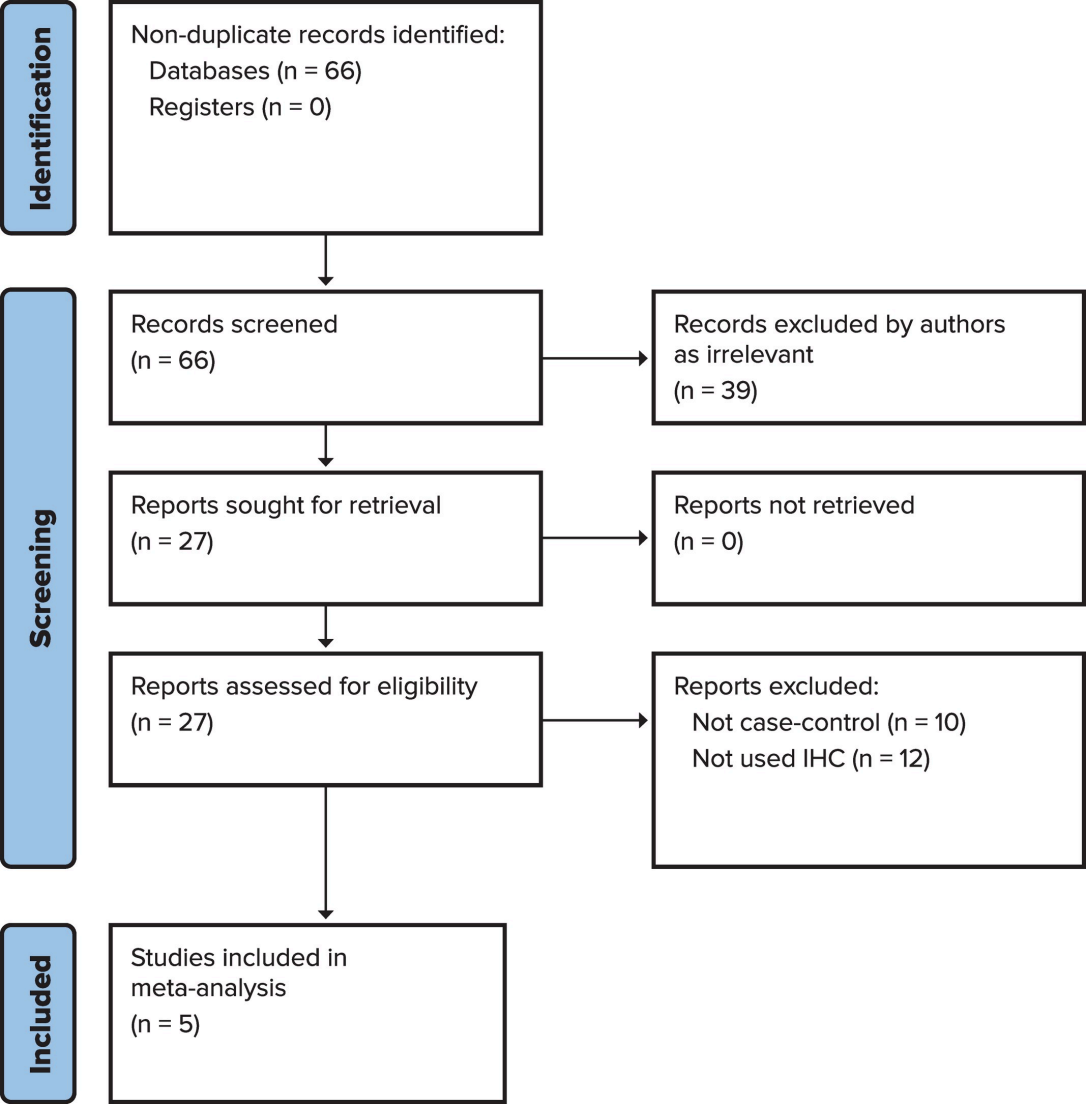
The PRISMA flow chart of study selection.

The literature search yielded 66 non-duplicate publications (Fig 1). Studies were excluded as irrelevant if they were reviews or letters to the editor (n = 14); not in humans (n = 1); not in English (n = 1); had different hypotheses (n = 11); different methods or different histologic cases (n = 7); studying seroreactivity or immune response (n = 5). Twenty-seven eligible studies reported the prevalence of JCV DNA in cases and controls by PCR or nested-PCR, and/or JCV T-antigen protein expression by IHC. During data extraction 10 studies were excluded for not using controls and 12 studies for not using IHC to identify JCV T-antigen protein in their analysis. Only five studies that reported JCV DNA by PCR and analysed the JCV DNA positive tissues by IHC for T-antigen protein expression were used for the meta-analysis. Therefore, five publications were eligible for analysis (Fig 1) [40, 60–63]. The characteristics of eligible studies are summarized in Table 1. Table 2A shows four case-control studies included in the analysis from colorectal and adenoma cancer patients. Selgrad et al. conducted IHC analyses in two separate populations in their study with adenoma cases in liver transplant patients (LTR) and non-LTR patients. In our analysis we also used those results as two separate populations, the LTR patient population and the non-LTR patient population (Table 2B) [40]. Hence, our meta-analysis includes six separate populations analyzed from five studies included and the data extracted are shown in Table 2A and 2B. The five studies included in the meta-analysis are published between 2003 and 2008 with two conducted in Europe, one in the United States, and two in Asia. Most studies reported that age did not differ significantly between cases and controls. Table 2 describes which controls were used in each study. Surgically or endoscopically resected specimen were obtained prospectively in 1 of the 5 studies, 3 studies selected random samples from pathology archives, databases, or registries, and 1 study did not specify. Table 2 summarizes the prevalence of the T-antigen protein expression in these studies and their calculated ORs. Two studies did not find T-antigen expression in either cases or controls (Table 2).

**Table 1:** Summary of basic characteristics of the 5 case-control studies included in meta-analysis for JCV T-antigen protein expression by IHC in CRC or adenoma.

Author, Year	^e^NOS	Country	n Case	n Control	Case tissue histology	Control tissue type	Methods	DNA integrity control	^i^Topo I	Target protein	Sample
Losa HJ, 2003	6	Spain	100	100	^a^CRC	normal mucosa (unspecified type)	nested ^g^PCR; ^c^IHC;	^f^NR	Yes ^i^TISPA	T-antigen	Frozen
Hori R, 2005	8	Japan	23 and 21	20	CRC and adenomas, resp	non-CRC (healthy colonoscopy)	nested PCR, IHC;	^b^GAPDH	No	T-antigen; VP, agnoprotein;	^h^PET
Goel A, 2006	6	USA	100	25	CRC	matched distal normal	PCR, IHC; Sequencing;	NR	NR	T-antigen	PET
Lin PY, 2008	6	Taiwan	22	22	CRC (adenocarcinomas)	matched adjacent normal	nested PCR; Sequencing; IHC;	NR	NR	The constant regulatory region (PCR); T-antigen, VP1 (IHC)	PET
Selgrad M, 2008	8	Netherlands	40 and 26	21 and 15	adenomas (non-^c^LTRs and LTRs, resp.)	non-CRC (healthy colonoscopy, non-LTRs and LTRs, resp.)	PCR; IHC;	NR	NR	T-antigen	PET

List of abbreviations.

^a^CRC = colorectal cancer.

^b^GAPDH = human glyceraldehyde-3-phosphate dehydrogenase gene.

^c^IHC = Immunohistochemistry.

^d^LTR = liver transplant recipients.

^e^NOS = Newcastle-Ottawa Scale (Quality control scale out of 10 points).

^f^NR = not reported.

^g^PCR = Polymerase chain reaction.

^h^PET = Paraffin embedded tissue.

^i^Topo I = Topoisomerase I used; TISPA = Topoisomerase I-sensitive polyomavirus amplification.

**Table 2:** JCV T-antigen protein expression by IHC, Odds ratios in cases vs. controls.

**Author, Year (analyses)**	**Cases**	**Controls**	**OR**	**95% CI**	***p* value**
Losa 2003 normal tissue controls	0% (0/100)	0% (0/100)	1	0.0196–50.8937	1
Hori 2005 non-CRC controls	0% (0/23)	0% (0/20)	0.8723	0.0166–45.9671	0.9462
Goel 2006 distal normal site controls	43% (43/100)	0% (0/25)	38.5826	2.2849–651.5010	0.0113
Lin 2008 matched adjacent normal	63.6% (14/22)	0% (0/22)	76.7647	4.1089–1434.1717	0.0037
**2B: Adenoma cases vs. controls of matched adjacent normal tissue**					
**Author, Year (analyses)**	**Cases**	**Controls**	**OR**	**95% CI**	***p* value**
Selgrad 2008 LTR patients (1)	50% (13/26)	0% (0/15)	31	1.6795–572.2022	0.021
Selgrad 2008 non-LTR patients (2)	5% (2/40)	0% (0/21)	2.7922	0.1281–60.8714	0.5137

(A)JCV T-antigen protein expression in CRC patient cases vs. normal tissue (within the same patient) or non-CRC (healthy patients) controls, as specified. (B)JCV T-antigen protein expression in LTR and non-LTR adenoma patient cases vs. normal tissue (within the same patient) controls.

List of abbreviations.

^a^CI = Confidence intervals.

^b^CRC = colorectal cancer.

^c^LTR = Liver transplant patients

^d^OR = Odds ratios.

### JCV T-antigen protein expression by immunohistochemistry, odds ratios, and meta-analysis

Meta-analyses performed for studies using IHC assay for JCV T-antigen protein expression in cases and controls that were positive for JCV DNA by PCR. The antibodies used in these assays were the mouse monoclonal antibody against SV40 large T-antigen that cross-reacts with JCV T-antigen (clone PAb416). Three studies found JCV T-antigen expression much higher in tumour samples than in controls (matched adjacent normal tissues), of which the study by Goel et al. reported statistically significantly higher T-antigen protein expression in cases versus the distal normal site tissue controls (Table 2) [63]. The Lin et al. study did not report a p-value [61].

### JCV T-antigen protein expression in neoplasms (CRC and adenoma) versus controls

**Fig 2:**
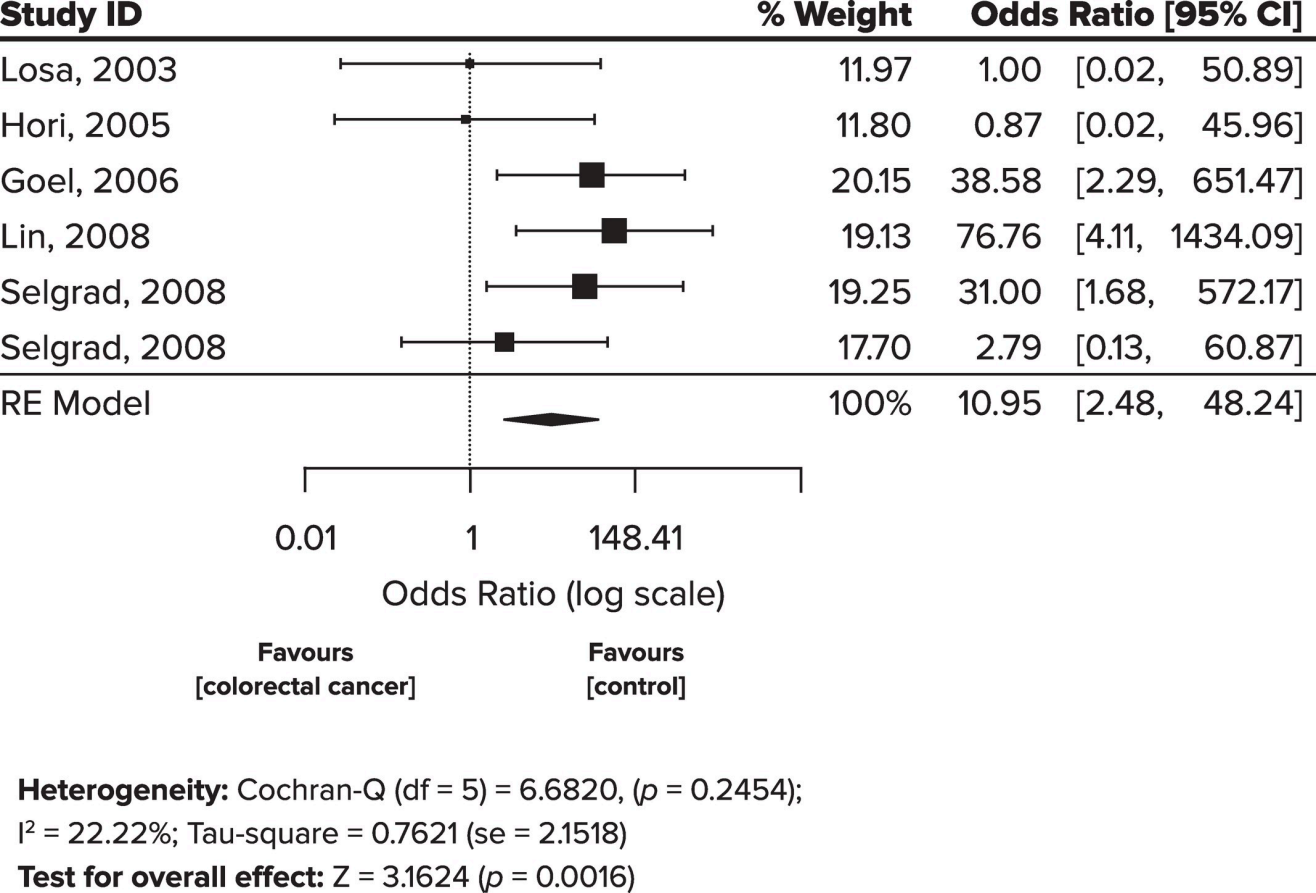
Effect of JCV T-antigen protein expression on the risk of neoplasm development (vs. any control tissue). Meta-analysis (RE = random-effects model) for the association between JCV T-antigen protein expression and the risk of neoplasm development in CRC and adenoma cases vs. controls.

Meta-analysis of all included studies (Table 2) was conducted to analyze the strength of a risk of JCV T-antigen protein expression and neoplasm development (CRC and adenoma). This meta-analysis showed a significant risk between JCV T-antigen protein expression and neoplasm development, which was 10.95-fold higher for tissues with JCV T-antigen protein expression versus controls (OR 10.95, 95% CI: 2.48–48.24, P = 0.0016) (Fig 2). Heterogeneity is low in this analysis (I-squared = 22.22%), 95% CIs are overlapping and the test for overall effect has a significant Z = 3.1624 value (Fig 2). Since the i^2^ was low (i^2^ = 22.22%), fixed effects analysis was also conducted, however, the effect was similar.

### Publication bias and quality assessment of studies

**Fig 3:**
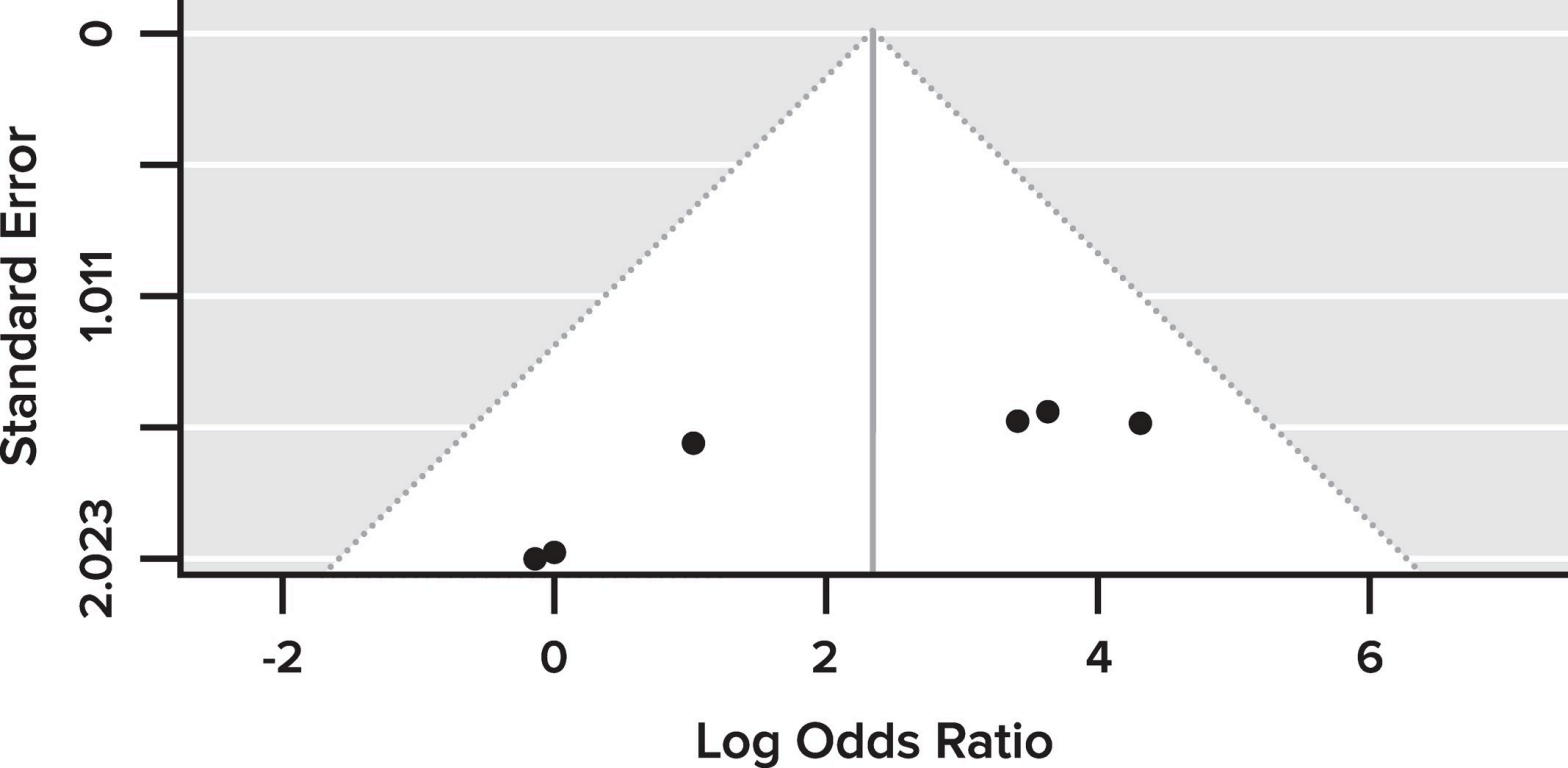
The funnel plot for the assessment of publication bias for the 5 studies included in the meta-analysis.

There was a low probability of publication bias based on the visualization of the funnel plot, which did not reveal a significant asymmetry (Fig 3). Studies were assessed for quality based on the Newcastle-Ottawa scale for case-control studies in which a study is rated by a ‘star system’ for a score of 1–10 on three broad perspectives [52]. The five studies included in the meta-analysis rated between 6 to 8 for quality (Table 1). Three studies rated 6 and two studies rated 8. Studies lost scores for lack of healthy (non-CRC or non-adenoma) subject controls, as most studies used healthy adjacent tissues as controls, not using additional criteria for control selection, lack of blinding, and lack of independent validation.

## Discussion

To our knowledge, this is the only meta-analysis on the association between JCV T-antigen protein expression and colorectal cancer development using all up-to-date published case-control studies. Progress in establishing the infectious origin of human cancers has been slow and controversial despite the evidence that infection plays a role in many naturally occurring cancers in animals [6, 64–66]. Studies report that 16% of all human cancers have an infectious origin and the most recent update estimates it at 20%, especially in the gastrointestinal tract [10, 11].

In this meta-analysis of crude ORs calculated from 5 case-control studies, we found that JCV T-antigen protein expression was significantly associated with increased risk of CRC and adenomas development. Selgrad et al. used two separate populations of LTR and non-LTR patients for their IHC analysis, hence our meta-analysis contains six distinct populations from five published studies [40]. Tissues were tested for T-antigen protein expression in JCV DNA positive samples. In the studies included the prevalence of JCV T-antigen was lower in case-control studies published in 2003–2005 than those in 2005–2008 where two studies failed to find any T-antigen protein expression in either cases or controls (Table 2). These two studies had also failed to find JCV DNA in their samples with Losa et al. showing none in either cases or controls and Hori et al. finding 6/23 in cases and 0/20 in controls [60, 62]. Since JCV T-antigen protein expression can only be tested in JCV DNA positive samples, in other words samples in which the virus was detected by PCR, technical difficulties such as poorly designed PCR primers or degradation of DNA samples in PET may have been a factor in the early studies for JCV DNA detection [25, 62, 67, 68]. Hori et al. commented that fixing samples in the preparation process may have been a factor in the subsequent immunochemistry for JCV T-antigen detection [60]. In addition, low DNA concentrations can limit the ability of conventional PCR to detect JCV due to the tight supercoiled topology of its viral DNA [20, 25]. Evidence, detecting JCV in both cases and controls came from later studies [10, 33, 60, 61, 69–74] that used JCV-specific primers, DNA quality control, and topoisomerase I pretreatment, which preferentially relaxes underwound or negatively supercoiled DNA [20, 25, 69]. These studies suggested an association between JCV and CRC with five reporting statistically significant increase of JCV DNA frequency in cases. JCV DNA was found in all patients who had gastrointestinal cancers and also in healthy controls (24–40%) [34]. Only one of the 5 studies reported the use of topoisomerase I treatment to improve JCV DNA detection by PCR, which represents a significant variable in the point estimates between the studies as JCV DNA prevalence may be underestimated. The importance of using topoisomerase I treatment and the use of proper technique to detect JCV DNA in samples was highlighted in the study by Langhi et al. as well as by Ricciardiello et al., who reported 27.9–75.8% frequency of JCV DNA detection in tissues depending on the type of technique used [25, 34]. Laghi et al. and Ricciardello et al. studies reported that treating the extracted DNA samples with topoisomerase I prior to PCR, which relaxes the supercoiled JCV dsDNA, yielded higher JCV DNA [20, 25, 34]. Newcomb et al. argued that topoisomerase I treatment was not necessary since JCV would be incorporated into chromosomal DNA [67]. It has been reported that polyoma viruses exist as episomal, plasmid like, dsDNA circles, and do not always exist as latent viruses [22, 25]. Hence, topoisomerase I treatment may be beneficial to detecting this virus in samples, which Ricciardello et al. noted as necessary for consistent detection of JCV DNA [34]. Episomal existence is also true for HPV, and these viruses may exist in different forms depending on tissue histology of the sample [8, 75]. Tightly wound episomal viral form may not be as detectable as the latent viral existence without the topoisomerase I treatment. Therefore, detection methods may account for some JCV prevalence variability between the studies. JCV is known to mutate within a patient and between patients, however, the T-antigen sequences are highly conserved [21]. Therefore, this is unlikely to account for variance between JCV detection among the studies as all of them targeted the T-antigen DNA and subsequently its protein expression in the JCV DNA positive samples. Primers used to detect JCV DNA among studies varied considerably and this may account for additional variability. Future studies could also improve on their quality by using community controls versus matched normal samples from the same patient.

As noted by Bae and Kim, different types of control tissue can cause heterogeneity in the assessment of an association between cancer and infection [46]. However, we conducted our meta-analyses including studies with CRC and adenoma due to lack of studies. This may account for additional variability. Including more studies as they become available would be beneficial for precision.

Recent meta-analyses showed a significant association between the presence of JCV DNA in cases versus controls [47–49]. Only one meta-analysis conducted T-antigen protein expression analysis, however, with two studies included in their analysis was conducted only on prevalence and not in case-control studies [49]. We conducted our meta-analysis on five available case-control studies.

Since JCV DNA is found in normal tissue as well as in neoplastic tissue, albeit at different levels, Selgrad et al. asserted that JCV T-antigen protein expression would be a more convincing implication of JCV’s role in carcinogenesis than just JCV DNA alone [40]. Considering the effect JCV T-antigen protein has on cell transformation leading up to carcinogenesis, it was important to assess the association between T-antigen protein expression for the risk of CRC and adenoma development. It was not possible to avoid heterogeneity by conducting the analysis according to control tissue type due to the low number of studies available. Our meta-analysis assessing the risk in CRC cases versus controls (matched normal tissue) showed a significant association between T-antigen protein expression and neoplastic development (OR 10.95; 95% CI: 2.48–48.24; p = 0.0016) (Fig 2). Heterogeneity was low as interpreted by i^2^ = 22.22%, although the confidence intervals were wide. We retained the random-effects model as the fixed-effects model did not alter the results. The signal in this meta-analysis indicates an important finding that needs to be confirmed. It should be noted that all controls in the IHC studies showed 0% T-antigen protein expression versus cases [40, 60–63]. This is significant as all the controls tested by IHC were positive for JCV DNA suggesting that an immunosuppressive event took place in cases as JCV is known to re-activate under immunosuppression. Specifically, Selgrad et al. showed that T-antigen protein expression in LTR patients, which have undergone immunosuppressive treatment, is significantly higher than in non-LTR patients: 50% vs. 5% (*P* = 0.0002) [40]. The 50% T-antigen protein expression in LTR patients is similar to the T-antigen protein expression in CRC patients (43–63.6%), suggesting that a similar level of immunosuppression may be taking place [40, 61, 63]. Since all population is known for high JCV prevalence (90%+), the testing for JCV T-antigen protein expression, and not just JCV DNA presence, to assess carcinogenicity is important [29–33, 40].

There is evidence that immunosuppression plays a role in JCV pathogenesis [9, 21, 32, 36, 40]. JCV is known to be re-activated under immunosuppression in HIV/AIDS and multiple sclerosis patients causing a lytic infection (PML) in the oligodendrocytes in the brain, JCV permissive cells [9, 21, 32, 36]. The immunosuppressive regimens received by LTR patients in Selgrad were cyclosporine A, prednisolone, and azathioprine (n = 28), prednisolone and tacrolimus (n = 8), and prednisolone plus azathioprine and tacrolimus (n = 5) [40]. Since IHC for T-antigen protein expression is only possible in JCV DNA positive tissues, this important finding may indicate that T-antigen protein expression has taken place in tissues with an unknown immunosuppressive event.

A review by Gallia et al. points out that JCV is neurotropic as well as lymphotropic, and probably crossing the blood-brain barrier in infected B-lymphocytes [32]. The study by Casini et al. showed that JCV was also present in resident lymphocytes, and in some cases exclusively [33]. This could represent a possible source of JCV infection within the body and the brain [32].

Polyomaviruses are known to express T-antigen protein, most of which are capable of binding and inactivating p53 and pRb, the tumour suppressor proteins, including JCV and SV40 [21, 64]. The amount of T-antigen determines the degree of cell transformation [21] Selgrad et al. and Goel et al. found the JCV T-antigen protein expression exclusively in the nuclei [40, 63]. This was consistent with Rizzo, who also found SV40 T-antigen expression predominantly in the nuclei [22]. The study by Goel et al. found that JCV T-antigen protein expression, but not JCV DNA presence, to be significantly associated with aberrant methylation of multiple tumour-suppressor genes in CRC, which could silence their expression [18, 63]. The work of Goel et al. also indicated that JCV may play a key role in all 3 types of genetic and epigenetic instability found in CRC (aberrant methylation, loss of heterozygosity and microsatellite instability) [63]. JCV non-permissive cells allow translation of JCV proteins, except for the VP1 capsid protein, which was not found in any of the studies that analyzed for it in literature [16]. This observation is consistent with the fact that JCV does not cause lytic infection in colorectal tissue for which the VP1 protein would be a necessary component to produce complete JCV virions ready for lytic release.

Three studies in literature reported on JCV viral loads [69, 70, 71]. Statistically significant higher viral loads were present in CRC cases versus controls. Mou et al. reported that the absolute copy numbers for JCV DNA in CRC tumours were lower than one copy per cell [70]. This may be in line with the fact that CRC cells are not permissive to JCV lytic replication and spread [16, 21, 39]. It could also indicate that JCV in the colon tissue spreads cell to cell one copy at a time, either as one episome or a copy of a latent virus in the chromosomal DNA.

### Future research and limitations

Large prospective case-control and cohort studies with age-adjusted multivariate analyses (according to tumour histology and tumour differentiation for example) with consistent methods (including topoisomerase I treatment) are needed. The goal is to confirm that JCV DNA with JCV T-antigen protein expression under immunosuppression are implicated in CRC development. Research into discovering which immunosuppressive agents or events have the highest impact on JCV re-activation is also important. Potentially, anti-JCV-T-antigen protein antibodies could be researched as possible CRC treatment in the future as well as developing anti JCV vaccines for CRC prevention. Much is still to be learned about JCV and its role in cancer development. However, it seems that JCV research may be following in HPV’s footsteps into the future [9, 75]. This analysis is limited by lack of studies, small patient numbers, and differences in techniques in the early versus later studies. This could cause an underestimate in the size effect. Different JCV DNA frequencies in samples and controls could be due to different patient populations tested and the JCV studied representing a different JCV type variant (Table 1) [21]. This needs further investigation and could be reminiscent of the same behaviour by HPV [9].

## Conclusion

The results of this first meta-analysis of JCV infection and the risk of CRC support the argument that JCV infection along with JCV T-antigen protein expression increases the risk of colorectal cancer. If confirmed in larger studies, knowing the risk associated with JCV infection and CRC development could be used to develop therapeutic and preventative measures, including vaccines, building on the success of the HPV vaccination developed to prevent cervical cancer [76].

## Supporting information

S1 Checklist PRISMA 2020 checklist. (DOCX)

## References

[1] World Health Organization [Internet]. Cancer. Updated September 2018. [updated 2022 Feb 3; cited 2022 May 1]; Available from: https://www.who.int/news-room/fact-sheets/detail/cancer

[2] World Cancer Research Fund International [Internet]. Worldwide cancer data. [updated 2022 Mar 23; cited 2022 May 1]; Available from: https://www.wcrf.org/.

[3] Cummins J, Tangney M. Bacteria and tumours: causative agents or opportunistic inhabitants? Infect Agent Cancer. 2013 Mar 28;8(1):11. doi: 10.1186/1750-9378-8-11 23537317 PMC3668256

[4] Chen H, Chen XZ, Waterboer T, et al. Viral infections and colorectal cancer: a systematic review of epidemiological studies. Int J Cancer. 2015 Jul 1; 137(1):12–24. doi: 10.1002/ijc.29180 25186851

[5] Collins D, Hogan AM, Winter DC. Microbial and viral pathogens in colorectal cancer. Lancet Oncol. 2011 May;12(5):504–12. doi: 10.1016/S1470-2045(10)70186-8 21067973

[6] Goff SP. Retroviridae. In: Knipe DM, Howley, editors. Fields virology. 6th ed. Philadelphia (PA): Lippincott Williams and Wilkins; 2013. p 1424–1473.

[7] Clifford GM, Smith JS, Plummer M, et al. Human papillomavirus types in invasive cervical cancer worldwide: a meta-analysis. Br J Cancer. 2003 Jan 13; 88(1):63–73. doi: 10.1038/sj.bjc.6600688 12556961 PMC2376782

[8] zur Hausen H. Papillomaviruses and cancer: from basic studies to clinical application. Nat Rev Cancer. 2002 May;2(5):342–50. doi: 10.1038/nrc798 12044010

[9] Maginnis MS, Atwood WJ. JC virus: an oncogenic virus in animals and humans? Semin Cancer Biol. 2009 Aug; 19(4):261–9. doi: 10.1016/j.semcancer.2009.02.013 19505654 PMC2694964

[10] Ksiaa F, Allous A, Ziadi S, et al. Assessment and biological significance of JC polyomavirus in colorectal cancer in Tunisia. J BUON. 2015 May-Jun; 20(3):762–9. 26214628

[11] Parkin DM. The global health burden of infection-associated cancers in the year 2002. Int J Cancer. 2006 Jun 15; 118(12):3030–44. doi: 10.1002/ijc.21731 16404738

[12] Hocker M, Hohenberger P. Helicobacter pylori virulence factors—one part of a big picture. Lancet. 2003 Oct 11;362(9391):1231–3. doi: 10.1016/S0140-6736(03)14547-3 14568748

[13] Hong SN, Lee SM, Kim JH, et al. Helicobacter pylori infection increases the risk of colorectal adenomas: cross-sectional study and meta-analysis. Dig Dis Sci. 2012 Aug; 57(8):2184–94. doi: 10.1007/s10620-012-2245-x 22669208

[14] Murphy G, Pfeiffer R, Camargo MC, et al. Meta-analysis shows that prevalence of Epstein-Barr virus-positive gastric cancer differs based on sex and anatomic location. Gastroenterology. 2009 Spet; 137(3):824–33. doi: 10.1053/j.gastro.2009.05.001 19445939 PMC3513767

[15] Buck CB, Van Doorslaer K, Peretti A, et al. The ancient evolutionary history of polyomaviruses. PLoS pathog. 2016 Apr 19; 12(4):e1005574. doi: 10.1371/journal.ppat.1005574 27093155 PMC4836724

[16] Ahye N, Bellizzi A, May D, et al. The role of the JC Virus in central nervous system tumorigenesis. Int J Mol Sci. 2020 Aug 28;21(17):6236. doi: 10.3390/ijms21176236 32872288 PMC7503523

[17] Padgett B, Zurhein G, Walker D, et al. Cultivation of papova-like virus from human brain with progressive multifocal leucoencephalopathy. Lancet. 1971 Jun 19;1(7712):1257–60. doi: 10.1016/s0140-6736(71)91777-6 4104715

[18] Shivdasani R.A. Molecular Biology of colorectal cancer. In: DeVita VT, Lawrence TS, Rosenberg SA, editors. Cancer: principles & practice of oncology. 9th ed. Philadelphia (PA): Lippincott Williams & Wilkins, 2012. p. 1074–1083.

[19] Khalili K, Del Valle L, Otte J, et al. Human neurotropic polyomavirus, JCV, and its role in carcinogenesis. Oncogene. 2003 Aug 11;22(33):5181–91. doi: 10.1038/sj.onc.1206559 12910255

[20] Ricciardiello L, Chang DK, Laghi L, et al. Mad-1 is the exclusive JC virus strain present in the human colon, and its transcriptional control region has a deleted 98-base-pair sequence in colon cancer tissues. J Virol. 2001 Feb;75(4):1996–2001. doi: 10.1128/JVI.75.4.1996-2001.2001 11160700 PMC115147

[21] DeCaprio JA, Imperiale MJ, Major EO. Polyomaviruses. In: Knipe D, Howley P, editors. Fields virology. 6th ed. Philadelphia (PA): Lippincot Williams & Wilkins; 2013. p. 1633–1661.

[22] Rizzo P, Bocchetta M, Powers A, et al. SV40 and the pathogenesis of mesothelioma. Semin Cancer Biol. 2001 Feb; 11(1):63–71. doi: 10.1006/scbi.2000.0347 11243900

[23] Chesters PM, Heritage J, McCance DJ. Persistence of DNA sequences of BK virus and JC virus in normal human tissues and in diseased tissues. J Infect Dis. 1983 Apr; 147(4):676–84. doi: 10.1093/infdis/147.4.676 6302172

[24] White MK, Gordon J, Khalili K. The rapidly expanding family of human polyomaviruses: recent developments in understanding their life cycle and role in human pathology. PLoS pathog. 2013 Mar; 9(3):e1003206. doi: 10.1371/journal.ppat.1003206 23516356 PMC3597531

[25] Laghi L, Randolph AE, Chauhan DP, et al. JC virus DNA is present in the mucosa of the human colon and in colorectal cancers. Proc Natl Acad Sci U S A. 1999 Jun 22;96(13):7484–9. doi: 10.1073/pnas.96.13.7484 10377441 PMC22112

[26] Feng H, Shuda M, Chang Y, et al. Clonal integration of a polyomavirus in human Merkel cell carcinoma. Science. 2008 Feb 22; 319(5866):1096–100. doi: 10.1126/science.1152586 18202256 PMC2740911

[27] Enam S, Del Valle L, Lara C, et al. Association of human polyomavirus JCV with colon cancer: evidence for interaction of viral T-antigen and β-catenin. Cancer Res. 2002 Dec 1;62(23):7093–7101. 12460931

[28] Nosho K, Shima K, Kure S, et al. JC virus T-antigen in colorectal cancer is associated with p53 expression and chromosomal instability, independent of CpG island methylator phenotype. Neoplasia. 2009 Jan;11(1):87–95. doi: 10.1593/neo.81188 19107235 PMC2606122

[29] Major EO, Neel JV. The JC and BK human polyoma viruses appear to be recent introductions to some South American Indian tribes: there is no serological evidence of cross-reactivity with the simian polyoma virus SV40. Proc Natl Acad Sci U S A. 1998 Dec 22;95(26):15525–30. doi: 10.1073/pnas.95.26.15525 9861002 PMC28076

[30] Padgett BL, Walker DL. Natural history of human polyomavirus infections. In: Stevens JG, editor. Persistent Viruses. Academic, New York (NY): Academic Press; 1978. p. 751–8.

[31] Dorries K. Molecular biology and pathogenesis of human polyomavirus infections. Dev Biol Stand. 1998;94:71–9. 9776228

[32] Gallia GL, Houff SA, Major EO, et al. Review: JC virus infection of lymphocytes—revisited. J Infect Dis. 1997 Dec; 176(6):1603–9. doi: 10.1086/514161 9395374

[33] Casini B, Borgese L, Del Nonno F, et al. Presence and incidence of DNA sequences of human polyomaviruses BKV and JCV in colorectal tumor tissues. Anticancer Res. 2005;25:1079–85. 15868949

[34] Ricciardiello L, Laghi L, Ramamirtham P, et al. JC virus DNA sequences are frequently present in the human upper and lower gastrointestinal tract. Gastroenterology. 2000 Nov;119(5):1228–35. doi: 10.1053/gast.2000.19269 11054380

[35] Monaco MC, Atwood WJ, Gravell M, et al. JC virus infection of hematopoietic progenitor cells, primary B lymphocytes, and tonsillar stromal cells: implications for viral latency. J Virol. 1996 Oct;70(10):7004–12. doi: 10.1128/JVI.70.10.7004-7012.1996 8794345 PMC190751

[36] Dalianis T, Hirsch HH. Human polyomaviruses in disease and cancer. Virology. 2013 Mar 15;437(2):63–72. doi: 10.1016/j.virol.2012.12.015 23357733

[37] White MK, Gordon J, Reiss K, et al. Human polyomaviruses and brain tumors. Brain Res Brain Res Rev. 2005 Dec 1;50(1):69–85. doi: 10.1016/j.brainresrev.2005.04.007 15982744

[38] Itoh S. [Analysis of the cellular tropism of human polyoma JC virus (JCV)]. Hokkaido Igaku Zasshi. 1995 Sept;70(5):765–74. 8543282

[39] Imperiale MJ. Oncogenic transformation by the human polyomaviruses. Oncogene. 2001 Nov 26;20(54):7917–23. doi: 10.1038/sj.onc.1204916 11753674

[40] Selgrad M, Koornstra JJ, Fini L, et al. JC virus infection in colorectal neoplasia that develops after liver transplantation. Clin Cancer Res. 2008 Oct 15;14(20):6717–21. doi: 10.1158/1078-0432.CCR-08-0961 18927316 PMC2846598

[41] Pagano JS, Blaser M, Buendia MA, et al. Infectious agents and cancer: criteria for a causal relation. Semin Cancer Biol. 2004 Dec;14(6):453–71. doi: 10.1016/j.semcancer.2004.06.009 15489139

[42] Bae JM, Kim EH. Epstein-Barr virus and gastric cancer risk: A meta-analysis with meta-regression of case-control studies. J Prev Med Public Health. 2016 Mr;49(2):97–107. doi: 10.3961/jpmph.15.068 27055546 PMC4829373

[43] Li N, Yang L, Zhang Y, et al. Human papillomavirus infection and bladder cancer risk: a meta-analysis. J Infect Dis. 2011 Jul 15;204(2):217–23. doi: 10.1093/infdis/jir248 21673031 PMC3114469

[44] Yang L, Xie S, Feng X, et al. Worldwide prevalence of human papillomavirus and relative risk of prostate cancer: A meta-analysis. Sci Rep. 2015 Oct 6;5:14667. doi: 10.1038/srep14667 26441160 PMC4594101

[45] Zhu H, Shen Z, Luo H, et al. Chlamydia trachomatis infection-associated risk of cervical cancer: A meta-Analysis. Medicine (Baltimore). 2016 Mar;95(13):e3077. doi: 10.1097/MD.0000000000003077 27043670 PMC4998531

[46] Bae JM, Kim EH. Human papillomavirus infection and risk of breast cancer: a meta-analysis of case-control studies. Infect Agent Cancer. 2016 Mar 14;11:14. doi: 10.1186/s13027-016-0058-9 26981149 PMC4791894

[47] Kimla LJ. John Cunningham virus infection and the risk of colorectal cancer: A meta analysis of case-control studies. American Society of Clinical Oncology. 2018 June 1–5; Chicago (IL); 2018. Abstract e15650.

[48] Shavaleh R, Kamandi M, Feiz Disfani H, et al. Association between JC virus and colorectal cancer: systematic review and meta-analysis. Infect Dis (Lond). 2020 Mar;52(3):152–160. doi: 10.1080/23744235.2019.1692145 31766929

[49] Shoraka HR, Abobakri O, Naghibzade Tahami A, et al. Prevalence of JC and BK viruses in patients with colorectal cancer: a systematic review and meta-analysis. Asian Pac J Cancer Prev. 2020 Jun 1;21(6):1499–1509. doi: 10.31557/APJCP.2020.21.6.1499 32592342 PMC7568898

[50] Haidich AB. Meta-analysis in medical research. Hippokratia. 2010 Dec;14(Suppl 1):29–37. 21487488 PMC3049418

[51] Page MJ, McKenzie JE, Bossuyt PM, et al. The PRISMA statement: an update guideline for reporting systematic reviews. BMJ. 2021; 372:n71. 33782057 10.1136/bmj.n71 PMC8005924

[52] Wells GA, Shea B, O’Connell D, et al. The Newcastle-Ottawa Scale (NOS) for assessing the quality of nonrandomized studies in meat-analyses [Internet]. Ottawa (CND): The Ottawa hospital, Dept of Epidemiology and Community Medicine, University of Ottawa; 2021 [updated 2021; cited May 20, 2022] Available at: http://www.ohri.ca/programs/clinical_epidemiology/oxford.asp.

[53] MedCalc Software Ltd. Odds ratio calculator from MedCalc software version 20.114 [Internet] 2022; MedCalc Software [updated 2022; cited May 20, 2022]. Available at: https://www.medcalc.org/calc/odds_ratio.php.

[54] Higgins JP, Thompson SG, Deeks JJ, et al. Measuring inconsistency in meta-analyses. BMJ. 2003 Sept 6;327(7414):557–60. doi: 10.1136/bmj.327.7414.557 12958120 PMC192859

[55] DerSimonian R, Kacker R. Random-effects model for meta-analysis of clinical trials: an update. Contemp Clin Trials. 2007 Feb;28(2):105–14. doi: 10.1016/j.cct.2006.04.004 16807131

[56] Mantel N, Haenszel W. Statistical aspects of the analysis of data from retrospective studies of disease. J Natl Cancer Inst. 1959 Apr;22(4):719–48. 13655060

[57] The R project for statistical computing [Internet]. 2022. [updated 2022 Jun 23; cited 2022 July 1]. Available at: https://www.r-project.org.

[58] Sedgwick P, Marston L. How to read a funnel plot in a meta-analysis. BMJ. 2015 Sept; 321:h4718. 10.1136/bmj.h4718 26377337

[59] Egger M, Davey Smith G, Schneider M, et al. Bias in meta-analysis detected by a simple, graphical test. BMJ. 1997 Sept 13;315(7109):629–34. doi: 10.1136/bmj.315.7109.629 9310563 PMC2127453

[60] Hori R, Murai Y, Tsuneyama K, et al. Detection of JC virus DNA sequences in colorectal cancers in Japan. Virchows Arch. 2005 Oct;447(4):723–30. doi: 10.1007/s00428-005-0014-3 16021515

[61] Lin PY, Fung CY, Chang FP, et al. Prevalence and genotype identification of human JC virus in colon cancer in Taiwan. J Med Virol. 2008 Oct;80(10):1828–34. doi: 10.1002/jmv.21296 18712832

[62] Losa JH, Fernandez-Soria V, Parada C, et al. JC virus and human colon carcinoma: an intriguing and inconclusive association. Gastroenterology. 2003 Jan;124(1):268–9. doi: 10.1053/gast.2003.50033 12512060

[63] Goel A, Li MS, Nagasaka T, et al. Association of JC virus T-antigen expression with the methylator phenotype in sporadic colorectal cancers. Gastroenterology. 2006 Jun;130(7):1950–1961. doi: 10.1053/j.gastro.2006.02.061 16762618

[64] DiMaio D, Fan H. Viruses, cell transformation, and cancer. In: Knipe DM and Howley PM, editors. Fields Virology. 6th ed. Philadelphia (PA): Lippincott Williams and Wilkins, Wolters Kluwer business, 2013. p. 153–188.

[65] Coffin J, Blomberg J, Fan H, et al. ICTV virus taxonomy profile: Retroviridae 2021. J Gen Virol. 2021 Dec;102(12):001712. doi: 10.1099/jgv.0.001712 34939563 PMC8744268

[66] Weiss AT, Klopfleisch R, Gruber AD. Prevalence of feline leukaemia provirus DNA in feline lymphomas. J Feline Med Surg. 2010 Dec;12(12):929–35. doi: 10.1016/j.jfms.2010.07.006 21036089 PMC11135535

[67] Newcomb PA, Bush AC, Stoner GL, et al. No evidence of an association of JC virus and colon neoplasia. Cancer Epidemiol Biomarkers Prev. 2004 Apr;13(4):662–6. 15066935

[68] Boland C, Laghi L, Ricciardiello L, et al. JC virus and human colon carcinoma: An intriguing and inconclusive association-Reply. Gastroenterology. 2003;124:269–270. 10.1053/gast.2003.50033 12512060

[69] Theodoropoulos G, Panoussopoulos D, Papaconstantinou I, et al. Assessment of JC polyoma virus in colon neoplasms. Dis Colon Rectum. 2005 Jan;48(1):86–91. doi: 10.1007/s10350-004-0737-2 15690663

[70] Mou X, Chen L, Liu F, et al. Prevalence of JC virus in Chinese patients with colorectal cancer. PLoS One. 2012;7(5):e35900. doi: 10.1371/journal.pone.0035900 22606241 PMC3350510

[71] Vilkin A, Ronen Z, Levi Z, et al. Presence of JC virus DNA in the tumor tissue and normal mucosa of patients with sporadic colorectal cancer (CRC) or with positive family history and Bethesda criteria. Dig Dis and Sci. 2012 Jan;57(1):79–84. doi: 10.1007/s10620-011-1855-z 21830098

[72] Coelho TR, Gaspar R, Figueiredo P, et al. Human JC polyomavirus in normal colorectal mucosa, hyperplastic polyps, sporadic adenomas, and adenocarcinomas in Portugal. J Med Virol. 2013 Dec;85(2):2119–27. doi: 10.1002/jmv.23705 24009184

[73] Sinagra E, Raimondo D, Gallo E, et al. Could JC virus provoke metastasis in colon cancer? World J Gastroenterol. 2014 Nov 14;20(42):15745–9. doi: 10.3748/wjg.v20.i42.15745 25400458 PMC4229539

[74] Aldrubi IFI A.A-MB, Mukhlis FA, et al. The Validity of Real Time PCR and in Situ Hybridization in the Detection of JCV in Colonic Biopsies of Patient with Colorectal Cancer. Int. J. Curr. Microbiol. App. Sci. 2015; 4(12):107–120.

[75] zur Hausen H. Papillomaviruses in the causation of human cancers—a brief historical account. Virology. 2009 Feb 20;384(2):260–265. doi: 10.1016/j.virol.2008.11.046 19135222

[76] Sankaranarayanan R. HPV vaccination. The most pragmatic cervical cancer primary prevention strategy. Int J Gynecol Obstet. 2015 Oct;131 Suppl 1:S33–S35. 10.1016/j.ijgo.2015.02.014 26433502

